# Day care cataract surgery in Central and Southern Italy: a multicentric survey

**DOI:** 10.1186/1472-6963-7-16

**Published:** 2007-02-01

**Authors:** Salvatore Cillino, Alessandra Casuccio, Francesco Di Pace, Francesco Pillitteri, Giovanni Cillino, Gaetano Lodato

**Affiliations:** 1 Department of Clinical Neuroscience, Section of Ophthalmology, University of Palermo (Italy), via Liborio Giuffrè, 13 – 90127 Palermo, Italy

## Abstract

**Background:**

Cataract day surgery has rapidly gained worldwide acceptance, because the new surgical techniques and costs are generally lower than those involved in ordinary hospitalization. Cataract surgery serves as a proxy indicator of the trend towards day surgery hospitalization in Italy and, therefore, of regional variability in health-care delivery and cost. The aim of this study was to update the diffusion of cataract day surgery through various surgical ophthalmological centers in central and southern Italy during 2005.

**Methods:**

A two-stage stratified cluster sampling method was used to draw a sample of Cataract Surgery Unit from Ophthalmic Units of central and southern Italy. A questionnaire was sent to 25 cataract surgery centers in nine health districts that represented the range of establishments (public, private, accredited or otherwise) in which cataract surgery is performed. Data were collected on numbers of procedures performed in 2005, hospital admission type, time from the onset of cataract day surgery, surgical procedure, and presence of other surgical centers.

**Results:**

The response rate was 42% (10 surveys), resulting in at least one completed questionnaire for each of these 9 districts. There is a positive trend towards day surgery hospitalization in all surgical centers. The percentage of patients treated as outpatients during 2005 varied from 50–60% (Avellino, Naples, Campobasso), to 80–90% (Rome, Bari), up to 90–100% (Catania, Palermo, Siracusa and Trapani), with an increasing trend in all the centers studied. Few differences were found in surgical procedures, and these were statistically insignificant.

**Conclusion:**

Our results confirm a positive trend towards day surgery in place of hospital inpatient admission for cataract surgery. This trend is expected to close the existing regional gap in Italy. Increased efficiency is an overriding need for the National Health Service in order to improve the rationalization of resources.

## Background

Ambulatory cataract surgery has increased rapidly, largely because of new surgical techniques and because increasing numbers of elderly patients and increased service and procedural costs have led to a change from inpatient to outpatient hospital admissions. In Italy, Day Surgery (DS) started in the early '80s but has not developed equally in all regions, mainly because of differences in local regulations and costs. Internationally, the "ambulatory/day surgery" problem is still widely debated [1,2] and this issue has led many countries to create specific guidelines which, in turn, have had economic effects related to the lower cost of ambulatory procedures compared to hospital ones [3-7]. Moreover, the economic advantage of the day surgery has been emphasized in a recent randomized clinical study [3], which reported lower costs for outpatients than for inpatients (1001 vs. 1218 Euros; p < 0.001). This difference in cost was largely due to the higher cost associated with an overnight stay in hospital (104 vs. 349 Euros; P < 0.001), while the costs of surgical intervention and of follow-up were similar in both patient groups [3]

Another point, tightly linked to the problem of outpatient versus inpatient surgical procedures, is the evaluation of appropriate hospitalization. Recently, an official Italian document concerning the "Definition of Essential Levels of Assistance" [8,9] identified forty-three DRG (Diagnosis Related Groups) at high risk of being inappropriate if used for inpatients [10,11]. Such an analysis shows a patchy distribution of inappropriateness for DRG selected as indicators, with high variability in the efficiencies of local health care systems and among local social-demographic conditions in the Italian National Health System [12,13]. In particular, "Cataract Surgery" has an admissibility threshold value for inpatient/outpatient of 46% [9] and constitutes a "proxy" indicator of appropriateness, because this procedure is considered internationally to be the most appropriate choice for most cases.

The aim of our study was to provide a more detailed and updated picture of the diffusion of DS for cataract surgery. A multicentric survey was performed to compare two different approaches to cataract surgery (inpatient vs outpatient) in facilities representing the health care institutions (public, private, accredited or otherwise) typical of Central and Southern Italy.

## Methods

National and regional data on inpatient/outpatient admissions during the period 1999–2003 were obtained from the "Hospital Discharge Report" database from the Ministry of Health [14].

A two-stage stratified cluster sampling method was used to draw a sample of Cataract Surgery Unit from Ophthalmic Units. In the first stage we sampled all the Ophthalmic Units of the leading cities of Italian regions involved in the study; in the second stage we surveyed the Ophthalmic Surgery Units working since 2000 in which it was possible to carry out both an ordinary recovery and a Day Surgery.

In 2005, a questionnaire [see Additional file 1] was sent to 25 Cataract Surgery Units in 9 health districts. The response rate was 42%, and at least one completed questionnaire was returned from each of these 9 districts.

Considering that Italy is composed of 20 regions, the regions and centers involved in this study were: Lazio (Rome – Accredited Private Institution, Ophthalmic Surgery Clinic with day surgery, 15 inpatient beds), Campania (Naples – University Public Hospital, ophthalmic center of reference to regional level with day surgery, ophthalmic first aid, 20 inpatient beds; Avellino – Public Hospital, ophthalmic center of reference to provincial level with day surgery, ophthalmic first aid, 16 inpatient beds), Molise (Larino, Campobasso – Public Hospital, ophthalmic center of reference to regional level with day surgery, ophthalmic first aid, 22 inpatient beds), Puglia (Bari – University Public Hospital, ophthalmic center of reference to regional level with day surgery, ophthalmic first aid, 24 inpatient beds), Sicily (Catania – University Public Hospital, ophthalmic center of reference to regional level with day surgery, ophthalmic first aid, 22 inpatient beds; Palermo – University Public Hospital, ophthalmic center of reference to regional level with day surgery, ophthalmic first aid, 26 inpatient beds; Siracusa – Public Hospital, ophthalmic center of reference to provincial level with day surgery, ophthalmic first aid, 16 inpatient beds; Trapani – Private Institution, Ophthalmic Surgery Clinic with day surgery, 8 inpatient beds). Among these centers, 40% were University Public Hospitals, 30% Public Hospitals and 30% Private Institutions.The chief medical officers of the Cataract Surgery Units completed the surveys. The items in the questionnaire mainly concerned:

(a) number of procedures for cataract surgery performed during 2005, and type of hospital admission (inpatient vs. outpatient);

(b) information about the time since the introduction of DS for cataract surgery, and the current trend compared to the years before it was introduced;

(c) information about perioperative management (e.g. antibiotic prophylaxis), blood tests, type of anesthesia, use of topical skin disinfectants, average time of hospitalization and postoperative follow-up;

(d) information about local facilities working in the same field, both public and private.

If no answer had been received within two weeks of sending the questionnaire, a new one was sent to urge the recipients to complete it.The questionnaire used for the survey had not been validated by previous studies and included a quite high number of open questions. Moreover, some questions were dichotomized. On the other hand this short and simple questionnaire (pilot questionnaire), which could represent every single local reality, was utilized because of the need to establish the number of surgical procedures performed and the typology of admission (inpatient, outpatient) to the hospital center. The other questions, which concerned the kind of anesthesia, infection prophylaxis before surgery, requests for preoperative blood surveys and the time of postoperative follow up, were included to confirm the typology of admission and adherence to standard guidelines for cataract surgery.

Moreover, because the survey data did not influence patient management and the issue being investigated is a matter of public record, ethical approval for the study was not required.

### Statistical analysis

Data were analyzed using Pearson's chi square test and Fisher's exact test for frequency analysis. All p-values were two-sided and p-values less than 0.05 were considered statistically significant. Analyses were conducted using Epi Info software, version 3.2.2, (Centers for Disease Control and Prevention, Atlanta) and Systat Software version 8.0 (Systat Inc., Evanston, IL).

## Results

The response rate was 42% (10 surveys), including at least one completed questionnaire from each of the 9 health districts.

**Figure 1:**
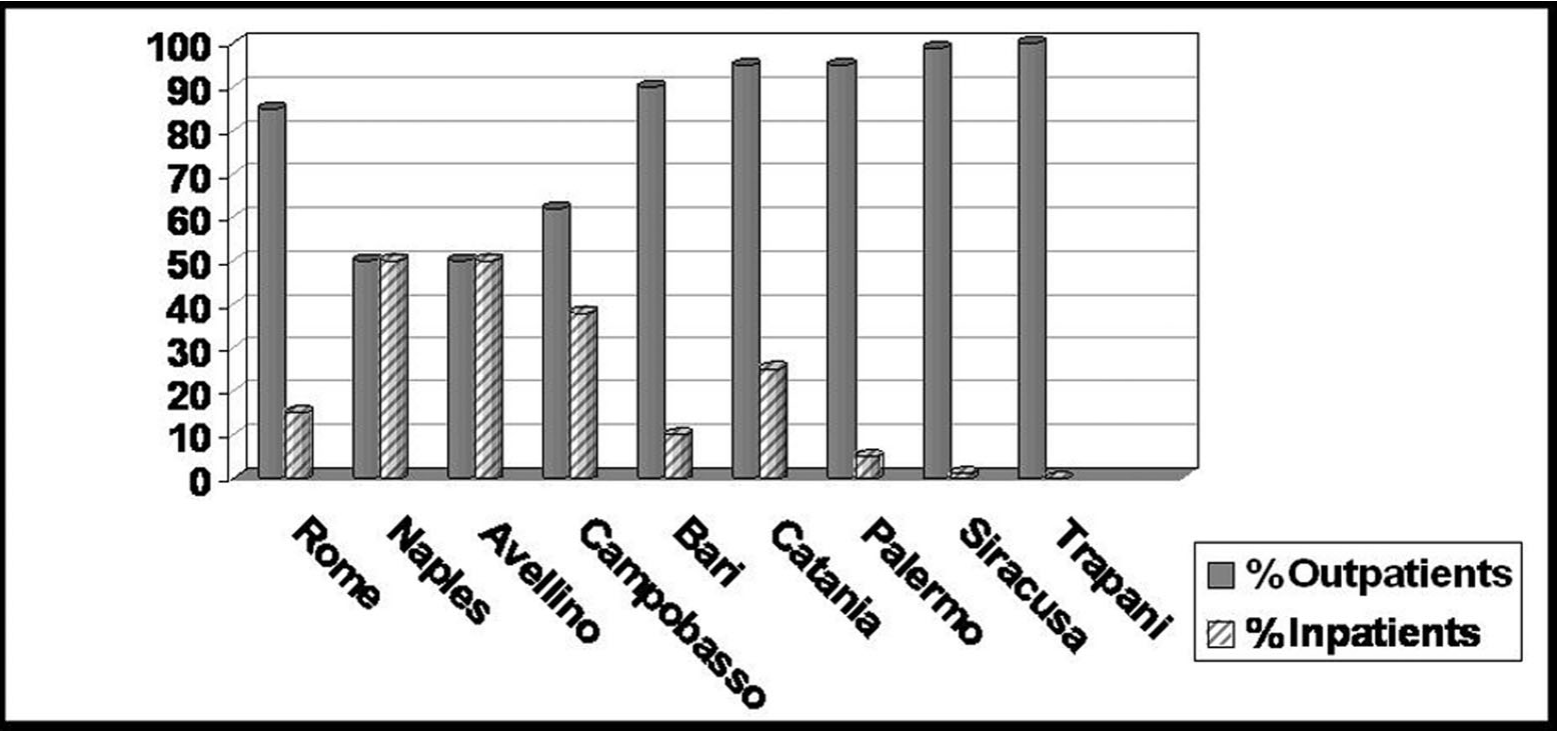
Percentage outpatients/inpatients for cataract surgery during 2005 in the centers studied in Central and Southern Italy.

The questionnaire responses showed that the number of surgical procedures for cataract in 2005 ranged from 350 in a Private Institution (Trapani) to 4,000 in a University Public Hospital (Bari) (Table 1). DS is active in all the facilities studied, and in particular it has been active for 2 years in Palermo and Siracusa, 3–4 years in Naples, Avellino, Rome and Bari, and more than 4 years in Trapani. The percentage of DS admissions compared to inpatients varied from 50–60% (Avellino, Naples, Larino-Campobasso), to 80–90% (Rome, Bari), up to 90–100% (Catania, Palermo, Siracusa and Trapani), with an increasing trend in all the centers studied (Figure 1).

**Table 1:** Data on number and type of surgical procedures and perioperative management for cataract surgery performed during 2005 in the centers studied in Central and Southern Italy

Center (Region)	Number of cataract surgery	Antibiotic prophylaxis	Preoperative laboratory test	Discharge time (hours)
Rome (Lazio)	1200	NO	YES	1
Avellino (Campania)	2500	YES	YES	2
Naples (Campania)	960	NO	YES	4
Larino-Campobasso (Molise)	2000	NO	YES	2
Bari (Puglia)	4000	YES	NO	2
Catania (Sicily)	1500	YES	YES	2
Palermo (Sicily)	1800	YES	YES	1
Siracusa (Sicily)	1760	YES	YES	2
Trapani (Sicily)	350	NO	YES	1

The surgical procedure data (Table 1) show that Rome, Naples, Larino (Campobasso) and Trapani perform no preoperative antibiotic prophylaxis, while Bari does not usually request preoperative laboratory tests. No center performs neuroleptanesthesia (just topical or peribulbar), and all centers perform a preoperative disinfection with povidone iodine (Table 1).

DS patients were usually discharged 1 to 4 hours after admission and inpatients after 24 hours. The follow-up was 24 hours in all centers except Bari (48 hours).

Statistical analysis showed no significant differences among types of institution (public or private, accredited or otherwise).

## Discussion

Internationally, there is an increasing diffusion of DS, especially for ophthalmic surgical procedures. In the USA, more than 2,200,000 cataract surgery procedures are performed annually on an ambulatory basis, and similar trends have been observed in many other countries by studies such as the International Association for Ambulatory Surgery (IAAS) [13,15-22].

In Italy, even in the absence of up-dated studies about the diffusion of DS for cataract surgery [25-28], national data [14] show a marked increase over recent years in the outpatient/inpatient admission rate for "Cataract Surgery" (27% in 1999, 38% in 2000, 47% in 2001, 62% in 2002 and 74% in 2003), especially in Northern and Central Italy but with some regional differences [9]. In particular, in the regions investigated in our survey, the outpatient/inpatient admission rates for cataract surgery obtained from the "Hospital Discharge Report" Database of the Ministry of Health [14] were: Lazio from 16% in 1999 to 72% in 2003; Molise from 0% in 1999 to 23% in 2003; Campania from 1% in 1999 to 46% in 2003; Puglia from 7% in 1999 to 55% in 2003; Sicily from 14% in 1999 to 75% in 2003. These rates and the observed trend agree with our results updated to 2005. Moreover, these data suggest that day cataract surgery rates are ubiquitously increasing but that there is still substantial variation among regional health districts.

The National Health Service in Italy is characterized by universal coverage and is currently financed through a regional tax on productive activities, general taxation collected centrally, various other regional taxes and users' copayments [23]. In 1992, the financing of hospital care was reformed, switching from a cost-reimbursement system to a prospective, activity-based payment system for inpatient and outpatient care. After 1995, hospitals came to be remunerated according to nationally predetermined rates based on DRGs. Regions are free to set lower DRG rates if they wish but must take the national rate as the maximum level [24]. As a result, funding rules for hospitals may differ significantly among regions. In the smallest Italian regions, the health department negotiates both volume and financing directly with the hospitals (which do not usually hold the status of trusts). In some southern regions, prospective funding based on DRGs has not yet been fully implemented [24].

Consequently, the differences in DS admission for cataract surgery are not related to the complexity of procedure. We think that because of health fund management at regional level, they are related instead to the socio-economic conditions typical of the South of Italy, including patient characteristics and differences in health politics, administrative approach and availability of budget for every single region and surgical units following the DRG classification [9,11].

The results of our survey, beyond confirming the increasing trend of DS admission in Central and Southern Italy compared to inpatient treatment, suggest that hospitals have not reached the 46% target [9,11] that the government set as the maximum threshold for inpatient admission. However, the 46% threshold represents just the first goal towards appropriate admissions. The main priority is to comply with standards already in use internationally, which are capable of reducing the acceptable threshold for inpatient admissions in cataract surgery from 46% to 25% [9]. On the other hand, we cannot consider the efficiency of a cataract surgery procedure only in terms of number of DS admissions, because we should pay more attention to the selection of ophthalmic patients, particularly since most of them are elderly (65 or over) and often have associated disease (cardiovascular or renal diseases, diabetes, etc.). For example, in our public hospital, about 1.2% of elderly patients had significant systemic complications postoperatively, and 45% of these required hospitalization. In some cases they may require assistance of a kind that is not typical of or suitable for ambulatory or day surgery, and appropriate clinical-managerial protocols may be needed, including, for example, more intensive postoperative checks [29].

However, as a possible limitation of our study, it was carried out at country level, and this unavoidably entails some imperfections. The use of day surgery within countries varies considerably and this variation may result from peculiar hospital or physician characteristics. Another limitation of our study was the relatively low response rate (42%), because of the lack of will among different hospitals to work together to find general behavioural attitudes, which precludes any consistent conclusion and allows only a descriptive analysis. The difficulty in recording all the units performing cataract surgical procedures is aggravated by the heterogeneity of health-care options in the same region, together with an insufficient level of information and an absence of relationship between the operative units. However, we have delineated the dispersal of economic funds due to inappropriate use of hospital admissions.

Moreover, as another limiting factor, the questionnaire used for the survey had not been validated by previous studies and has various drawbacks: it includes a quite high number of open questions and some questions are dichotomized. In consequence, the information obtained is usually inadequate and more detailed data are not provided. On the other hand, the aim of our survey was not to validate our questionnaire. Moreover, there is no validated questionnaire in the literature in this field of interest: the geographic variability of this survey hindered validation of any analysis and we chose a simple questionnaire (a pilot questionnaire) that could represent every single local reality.

## Conclusion

In conclusion, our study seems to confirm that the clinical approach to cataract surgery is similar at different sites, and we can therefore state that clinical and surgical issues do not influence the specificity of hospital admissions in any single region. Economic differences, different administrative approaches and the disposability of budget for every single region and for the single surgical units following the DRG Classification System represent the true limiting factors and the major concern.

The international literature does not report data comparable to our situation or to other countries, because of differing health politics and the peculiar relationship between citizen and National Health System in each country. Moreover, we must emphasize the importance of adopting day surgery rather than inpatient care for cost-effectiveness analysis and how much funding could be saved if all Italian regions adopted more day cataract surgery [30]. Some researches carried out in the USA and in other European countries, such as the UK, show that the move toward day surgery is associated with cost reductions ranging between 30% and 50% [31-33].

Whether a further increase of ambulatory surgery is desirable should ultimately be determined by further studies concerning the quality of care, patients' satisfaction and hospital appropriateness.

## Competing interests

The author(s) declare that they have no competing interests.

## Authors' contributions

SC conceived of the study and participated in its design and coordination.

AC participated in the design of the study and performed the statistical analysis.

FDP participated in the design and drafted the manuscript.

FP participated in the design of the study and helped to draft the manuscript.

GC participated in the design of the study and helped to draft the manuscript.

GL participated in the design and coordination of the study.

All authors read and approved the final manuscript.

## Pre-publication history

The pre-publication history for this paper can be accessed here:



## Supplementary Material

Additional file 1 **Questionnaire on cataract day surgery diffusion in Ophthalmic Surgery Unit of Central and Southern Italy**. The items in the questionnaire concerned: (a) number of procedures for cataract surgery performed during 2005, and type of hospital admission; (b) information about the time since the introduction of DS for cataract surgery; (c) information about perioperative management, blood tests, type of anesthesia, use of topical skin disinfectants, average time of hospitalization and postoperative follow-up; (d) information about local facilities working in the same field, both public and private. Click here for file

## References

[B1] International Association for Ambulatory Surgery – Council of Presidents (2001). Foundation and early history of the international association for ambulatory surgery 1995–2001. Ambulatory Surgery.

[B2] De Lathouwer C, Poullier JP (2000). How much ambulatory surgery in the World in 1996–1997 and trends?. Ambulatory Surgery.

[B3] Castells X, Alonso J, Castilla M, Ribò C, Cots F, Antò JM (2001). Outcomes and costs of outpatient and inpatient cataract surgery: a randomised clinical trial. J Clin Epidemiol.

[B4] Guzzanti E, Mastrobuono I (1999). Organisational, technological and structural standards for office based ambulatory surgery and day surgery. Ambulatory Surgery.

[B5] Kroneman MW, Westert GP, Groenewegen PP, Delnoij DMJ (2001). International variations in the availability and diffusion of alternatives to in-patient care in Europe: the case of day surgery. Ambulatory Surgery.

[B6] Nghiem-Buffet MH, de Pouvourville G, Renard G, Ullern M, Boureau C, Chaine G (2001). Cost of managing cataracts. Evaluation of traditional hospitalization and ambulatory surgery. Presse Med.

[B7] Ogg T (1998). Office-based surgery: how should the International Association for Ambulatory Surgery proceed?. Ambulatory Surgery.

[B8] Decree President Council of Ministers 29 November 2001 (2001). Definition of the Essential Levels of Assistance.

[B9] Ministry of Health (2002). Hospital discharge appropriateness in Italy with APPRO methodology Roma.

[B10] Cacciani L, Materia E, Cesaroni G, Baglio G, Davoli M, Arcá M, Perucci CA (2001). Income, income distribution and hospitalisation: an ecologic study in Rome, Italy. Health Policy and Economics: Strategic Issues in Health Care Management.

[B11] Materia E (2003). Appropriateness: origins, implications, evaluation. Tendenze Nuove.

[B12] Escarce JJ (1993). Would eliminating differences in physician practice style reduce geographic variations in cataract surgery rates?. Med Care.

[B13] Sanderson HF (1980). Regional variation in cataract extraction rates and their relationship with resource supply and need. J R Soc Med.

[B14] Ministry of Health: "Hospital Discharge Report" Database. http://www.ministerosalute.it/programmazione/sdo/ric_informazioni/default.jsp.

[B15] Galin MA, Boniuk V, Obstbaum S, Barasch KR, Baras I (1981). Hospitalization and cataract surgery. Ann Ophthalmol.

[B16] Davies PD, Limacher E, Powell K (1987). Outpatient cataract surgery 1982–1986. Eye.

[B17] Strong NP, Wigmore W, Smithson S, Rhodes S, Woodruff G, Rosenthal AR (1991). Day case cataract surgery. Br J Ophthalmol.

[B18] Davies B, Tyers AG (1992). Do patients like day case cataract surgery?. Br J Ophthalmol.

[B19] Holland GN, Earl DT, Wheeler NC, Straatsma BR, Pettit TH, Heppler RS, Christensen RE, Oye RK (1992). Results of inpatient and outpatient cataract surgery. A historical cohort comparison. Ophthalmology.

[B20] Percival SPB, Setty SS (1992). Prospective audit comparing ambulatory day surgery with inpatient surgery for treating cataracts. Quality Health Care.

[B21] Schein OD, Steinberg EP, Javitt JC, Cassard SD, Tielsch JM, Steinwachs JM, Legro MW, Diener-West M, Sommer A (1994). Variation in cataract surgery practice and clinical outcomes. Ophthalmology.

[B22] The Royal College of Ophthalmologists (1995). Guidelines for Cataract Surgery.

[B23] OECD (2002). OECD Health Data 2002", CD-ROM, Paris.

[B24] WHO (2002). "Health care systems in transition Italy", European observatory on health care systems.

[B25] Gamotis PB, Dearman VC, Doolittle NO, Price SC (1997). In-patient Versus Out-patient Satisfaction. Ambulatory Surgery.

[B26] De Lathouwer C, Poullier JP (1998). Ambulatory Surgery in 1994–1995: The state of the art in 29 OECD countries. Ambulatory Surgery.

[B27] Fedorowicz Z, Lawrence D, Gutierrez P Day care versus in-patient surgery for age-related cataract. Cochrane Database Syst Rev.

[B28] Italian Ophthalmological Society (S.O.I.) (1998). Clinical-organizational recommendations on cataract surgery.

[B29] Italian Ophthalmological Society (S.O.I) (2000). Guidelines on Cataract Day Surgery.

[B30] Siciliani L, Hurst J (2003). Explaining Waiting Times Variations for Elective Surgery across OECD Countries. OECD HEALTH WORKING PAPERS NO 7 DELSA/ELSA/WD/HEA.

[B31] Bloom B, Krueger N (1988). Cost and quality effects of outpatient cataract removal. Inquiry.

[B32] Steinberg EP, Javitt JC, Sharkey PD, Zuckerman A, Legro MW, Anderson GF, Bass EB, O'Day D (1993). The content and cost of cataract surgery. Arch Ophthalmol.

[B33] Aylward G, Larkin D, Cooling R (1993). Audit of cost and clinical outcomes of cataract surgery. Health Trends.

