# Supplementary material for: Day care cataract surgery in Central and Southern Italy: a multicentric survey
Source: BMC Health Serv Res. 2007 Feb 1;7:16. doi: 10.1186/1472-6963-7-16 (PMC1797011; doi:10.1186/1472-6963-7-16)
Supplement: Additional file 1 — Questionnaire on cataract day surgery diffusion in Ophthalmic Surgery Unit of Central and Southern Italy. The items in the questionnaire concerned: (a) number of procedures for cataract surgery performed during 2005, and type of hospital admission; (b) information about the time since the introduction of DS for cataract surgery; (c) information about perioperative management, blood tests, type of anesthesia, use of topical skin disinfectants, average time of hospitalization and postoperative follow-up; (d) information about local facilities working in the same field, both public and private. [file 1472-6963-7-16-S1.doc]

**Questionnaire on cataract day surgery diffusion in Ophthalmic Surgery Unit of Central and Southern Italy**

1. How many cataract procedures are performed in your facility? ………………............……
2. What is the percentage of day surgery and full hospitalization? ………….........................
3. Is cataract day surgery on the increase ? YES ……... NO ...………….
4. How long have you been performing cataract day surgery ? ……………………………….
5. Are there in your area mainly day surgery private/public facilities ? YES ….... NO ..........
6. Are there in your area mainly full hospitalization private/public facilities? YES ..... NO..….
7. Do patients receive infection prophylaxis before surgery ? YES …….. NO .………......
8. Are blood tests required before cataract surgery ? YES …….. NO .………......
9. What kind of anestesia is employed for cataract surgery ? .....……………………………..
10. How long after the surgery are patients discharged ? ……………………………………..
11. Do you perform a post-surgical follow-up and what are your follow-up stages ? YES ... NO ....

……………………………………..

……………………………………..

……………………………………..
